# Supplementary material for: Ischaemic preconditioning and pharmacological preconditioning with dexmedetomidine in an equine model of small intestinal ischaemia-reperfusion
Source: PLoS One. 2020 Apr 29;15(4):e0224720. doi: 10.1371/journal.pone.0224720 (PMC7190151; doi:10.1371/journal.pone.0224720)
Supplement: Attachment — Submitted filename: Response to Reviewers.docx [file pone.0224720.s002.docx]

Dear Editors, Dear Francsco, Dear Reviewers,

Thank you very much for taking your time to review our manuscript and making helpful comments. We tried to follow all required changes and additions as well as the required editorial adaptions.

All changes in the manuscript are highlighted in yellow.

Files were named as required

The source of horse is difficult to descibe as they are not purpose bred for experiments, I tried to add some information

The Ethics and funding statements have been adapted

In addition to the doctoral thesis, parts of this study were presented as an oral abstract and as poster abtract, with a peer review process and publication in conference proceedings. The abstracts comprise only a word count of less than 250 words and therefore, do not represent a dual publication. The abstracts are shown below:

I hope we could make all the required adaption.

With the best wishes, Sabine
